# Antidote availability in the municipality of Campinas, São Paulo, Brazil: Disponibilidade de antídotos no município de Campinas, São Paulo

**DOI:** 10.1590/1516-3180.2016.00171120816

**Published:** 2017-03-13

**Authors:** Luciane Cristina Rodrigues Fernandes, Taís Freire Galvão, Adriana Safioti Toledo Ricardi, Eduardo Mello De Capitani, Stephen Hyslop, Fábio Bucaretchi

**Affiliations:** I RN, MSc. Nurse, Campinas Poison Control Center, School of Medical Sciences, Universidade Estadual de Campinas (Unicamp), Campinas (SP), Brazil.; II BPharm, MSc, PhD. Professor, School of Pharmaceutical Sciences, Universidade Estadual de Campinas (Unicamp), Campinas (SP), Brazil, and Professor, Postgraduate Pharmaceutical Sciences Program, Universidade Federal do Amazonas (UFAM), Manaus (AM), Brazil.; III MD, MSc, PhD. Professor, Campinas Poison Control Center, and Professor, Department of Clinical Medicine, School of Medical Sciences, Universidade Estadual de Campinas (Unicamp), Campinas (SP), Brazil.; IV BSc, PhD. Professor, Campinas Poison Control Center, and Professor, Department of Pharmacology, School of Medical Sciences, Universidade Estadual de Campinas (Unicamp), Campinas (SP), Brazil.; V MD, MSc, PhD. Professor, Campinas Poison Control Center, and Professor, Department of Pediatrics, School of Medical Sciences, Universidade Estadual de Campinas (Unicamp), Campinas (SP), Brazil.

**Keywords:** Antidotes, Brazil, Poisoning, Strategic stockpile, Emergency medical services

## Abstract

**CONTEXT AND OBJECTIVE:**

The lack of availability of antidotes in emergency services is a worldwide concern. The aim of the present study was to evaluate the availability of antidotes used for treating poisoning in Campinas (SP).

**DESIGN AND SETTING:**

This was a cross-sectional study of emergency services in Campinas, conducted in 2010-2012.

**METHODS:**

The availability, amount in stock, place of storage and access time for 26 antidotal treatments was investigated. In the hospitals, the availability of at least one complete treatment for a 70 kg adult over the first 24 hours of admission was evaluated based on stock and access recommendations contained in two international guidelines.

**RESULTS:**

14 out of 17 functioning emergency services participated in the study, comprising pre-hospital services such as the public emergency ambulance service (SAMU; n = 1) and public emergency rooms for admissions lasting ≤ 24 hours (UPAs; n = 3), and 10 hospitals with emergency services. Six antidotes (atropine, sodium bicarbonate, diazepam, phytomenadione, flumazenil and calcium gluconate) were stocked in all the services, followed by 13 units that also stocked activated charcoal, naloxone and diphenhydramine or biperiden. No service stocked all of the recommended antidotes; only the regional Poison Control Center had stocks close to recommended (22/26 antidotal treatments). The 10 hospitals had almost half of the antidotes for starting treatments, but only one quarter of the antidotes was present with stocks sufficient for providing treatment for 24 hours.

**CONCLUSION:**

The stock of antidotes for attending poisoning emergencies in the municipality of Campinas is incomplete and needs to be improved.

## RESUMO

**CONTEXTO E OBJETIVO:**

A carência de disponibilidade de antídotos nas salas de emergência é uma preocupação mundial. O objetivo foi avaliar a disponibilidade de antídotos usados no tratamento de pacientes intoxicados no município de Campinas (SP).

**TIPO DE ESTUDO E LOCAL:**

Trata-se de estudo transversal de serviços de emergência de Campinas, realizado de 2010-2012.

**MÉTODOS:**

A disponibilidade, quantidade estocada, local de armazenamento e tempo de acesso a 26 tratamentos antidotais foi investigada. Nos hospitais, foi avaliada também a disponibilidade de pelo menos um tratamento completo para um adulto de 70 kg nas primeiras 24 horas da admissão, com base em recomendações de estoques e acesso contidas em duas diretrizes internacionais.

**RESULTADOS:**

14 dentre 17 serviços de emergência em funcionamento participaram do estudo, que incluiu serviços pré-hospitalares, como o Serviço de Atendimento Móvel de Urgência (SAMU, n = 1) e três Unidades de Pronto Atendimento (UPAs, internação limitada até 24 horas), além de 10 hospitais com emergência. Seis antídotos (atropina, bicarbonato de sódio, diazepam, fitomenadiona, flumazenil e gluconato de cálcio) estavam estocados em todos os serviços, seguidos de 13 que também estocavam carvão ativado, naloxona, difenidramina ou biperideno. Nenhum serviço tinha estoque de todos os antídotos recomendados; somente o Centro de Controle de Intoxicações regional tinha estoque próximo ao perfil recomendado (22/26 opções terapêuticas). Os 10 hospitais tinham quase metade dos antídotos necessários para iniciar tratamento, mas somente um quarto dos antídotos estava em estoques suficientes para oferecer tratamento por 24 horas.

**CONCLUSÃO:**

O estoque de antídotos para atendimento de emergências toxicológicas no município de Campinas é incompleto e deve ser melhorado.

## INTRODUCTION

The lack of adequate and readily available antidotes in emergency services is a worldwide concern.^1^^,^^2^^,^^3^^,^^4^^,^^5^^,^^6^^,^^7^^,^^8^^,^^9^ Various factors have been correlated with antidote unavailability, including product shelf-life, the high cost of certain antidotes, the classification of some antidotes as orphan drugs (i.e. drugs of low commercial interest that qualify for incentives such as tax relief, depending on each country’s policy) and difficulty in importing antidotes from certain countries.^8^

Guidelines may help to organize antidote stocks in emergency services in acute hospitals.^1^^,^^2^^,^^3^^,^^4^^,^^9^^,^^10^ The United Kingdom (UK) guidelines, which were initially published in 2008 and are regularly revised and updated, stipulate that antidote stocks should be sufficient for the initial treatment of a 70 kg adult patient and for treatment during the first 24 hours. In these guidelines, antidote stocks are also classified according to availability, i.e. antidotes held in emergency departments for immediate use, antidotes held in hospital pharmacies or dispensaries for use in emergency departments within one hour of patient admission, and antidotes that are rarely used or for which the time interval until use in emergency departments is not critical, with stocks held in a supraregional center.^3^^,^^10^

The United States (US) guidelines also classified antidotes based on their optimal access time.^4^ This list of antidotes and their minimum stocks was defined based on a systematic review of the literature followed by evaluation by a panel of experts, who assessed the efficacy, safety, influence of access time on subsequent treatment and dose required to treat a 100 kg-individual.^4^ Based on antidote guidelines, verification of antidote stocks in Canada had a beneficial impact on antidote availability in acute hospitals.^1^^,^^2^ In that context, two audits were held: one year before and one after the publication of antidote stockpile guidelines. In the second audit, conformity was found to have significantly increased, reaching at least 62% of the recommended stock for each antidote. To date, assessments of antidote availability in the Brazilian context are still lacking.

## OBJECTIVE

In this study, we assessed the availability of antidotes used to treat poisoning at the emergency services of the municipality of Campinas, in the state of São Paulo, southeastern Brazil. The analysis included both pre-hospital services and acute care hospitals.

## METHODS

### Study design and setting

This investigation was a cross-sectional study conducted among emergency services in Campinas, São Paulo, from April 2010 to April 2012. Campinas had an estimated population of 1.15 million inhabitants in 2014.^11^

We identified 17 emergency services registered in the National Registry of Healthcare Establishments of the Department of Information of the Brazilian National Health System (SUS): 10 private and seven public.^12^ We attempted to include the universe of eligible participants, and thus no sample size was predetermined.

### Participants

All emergency services in the municipality were eligible for this study: public pre-hospital services (the emergency ambulance service [Serviço de Atendimento Móvel de Urgência, SAMU] and emergency rooms with limited capacity for admission, i.e. admission for up to 24 hours [Unidade de Pronto Atendimento, UPA]); and any hospital that had inpatient beds and could be required to treat a poisoned patient (emergency hospitals, also referred to as acute hospitals).

### Variables

The primary endpoint was the frequency of antidotal treatment availability in the emergency service that was surveyed (pre-hospital or hospital). The secondary endpoints were the adequacy of the stockpile for the initial and subsequent 24 hours of treatment for an adult of 70 kg in the emergency departments.

We considered “antidote” to be one or more medicine that is appropriate for treating a case of poisoning. As a result, the list has more medicines than antidotes.

The variables of the institutions were their nature (public/private), complexity, number of beds and educational attainment of the pharmacy director. With regard to the antidotes, the variables were the availability of each medicine, amounts available in the main storage and in the emergency room, and time taken to make the medicine available in the emergency room (< 1 hour or ≥ 1 hour). The information on all the variables was given by the person responsible for the pharmacy of the emergency service, as described below.

### Data sources and measurement

The list of recommended antidotes was based on the UK (2008) and US (2009) guidelines (Table 1).^3^^,^^4^ We excluded the antidotes recommended for stocking in supraregional centers, since these do not need to be available in all emergency rooms. We also excluded phentolamine, which was recommended in only one guideline; and potassium iodide, because of lack of clinical demand within the Brazilian context.

**Table 1.** Pharmaceutical preparations and recommended stock for the antidotes evaluated in this study based on sufficient amounts for the initial and subsequent 24 hours of treatment of a 70 kg adult patient in the emergency room

Item	Antidote, route	Pharmaceutical preparation	Recommended stockpile	Clinical indication^a^
1	Acetylcysteine, IV	100 mg/ml; 3 ml ampoule	70 ampoules	Paracetamol
	Acetylcysteine, PO	600 mg sachet	155 sachets	
2	Activated charcoal, PO	10 g, 25 g or 50 g sachets	300 g (e.g., 6 x 50 g sachets)	Adsorbent for gastrointestinal decontamination
3	Anti-digoxin antibodies, IV	38 mg vial	10 vials	Cardioactive steroids
4	Atropine, IV	250 µg/ml; 1 ml ampoule	300 ampoules	Cholinesterase inhibitors
5	Calcium folinate, IV	300 mg vial; 50 mg vial; 3 mg/ml; 1 ml ampoule	3 x 300 mg vials, or 16 x 50 mg vials, or 240 ampoules	Methotrexate; methanol, formic acid
6	Calcium gluconate, IV	10% (100 mg/ml); 10 ml ampoules	12 ampoules	Calcium channel blockers, hydrofluoric acid
7	Calcium gluconate, topic	gel 2.5%; 25 g pack	1 pack	Hydrofluoric acid burns
8	Dantrolene, IV	20 mg vial	35 vials	Neuroleptic malignant syndrome
9	Desferrioxamine, IV	500 mg vial	12 vials	Iron salts
10	Diazepam, IV	5 mg/ml; 2 ml ampoule	4 ampoules	Convulsions, agitation and precordial pain
11	Dimercaprol, IM	100 mg/ml; 1 ml ampoule	15 ampoules	Mercury, arsenic, gold
12	Diphenhydramine, IV	50 mg/ml; 1 ml ampoule	4 ampoules	Dystonic reactions
	Biperiden, IV	5 mg/ml; 1 ml ampoule	4 ampoules	
13	Flumazenil, IV	100 µg/ml; 5 ml ampoule	4 ampoules	Benzodiazepines
14	Fomepizole, IV	5 mg/ml; 20 ml ampoule	25 ampoules	Methanol, ethylene glycol
	Ethanol, IV	100%; 10 ml ampoule	30 ampoules	
15	Glucagon, IV	1 mg vial	50 vials	Beta blockers, calcium channel blockers, tricyclic antidepressants
16	Hydroxocobalamin, IV	5 g pack	2 packs	Cyanide
	Sodium nitrite, IV and	3% (30 mg/ml); 10 ml ampoule	1 ampoule	
	sodium thiosulfate, IV	25% (250 mg/ml); 10 ml ampoule	8 ampoules	
17	Methylene blue, IV	1-2 mg/ml; 5 ml ampoule	3-6 ampoules	Methemoglobin-inducing agents
18	Naloxone, IV	0.4 mg/ml; 1 ml ampoule	25 ampoules	Opioids
19	Octreotide, IV	0.1 mg/ml; 1 ml ampoule	2 ampoules	Oral hypoglycemic agents
20	Physostigmine, IV	1 mg/ml; 2 ml ampoule	2 ampoules	Anticholinergic agents
21	Phytomenadione (vitamin K), IV	10 mg/ml; 1 ml ampoule	1 ampoule	Coumarin anticoagulants
22	Polyethylene glycol 3350, PO	Sachets, reconstituted with water (2 l)	12 sachets	Iron salts, lithium, packs of cocaine or heroin (body packers)
23	Pralidoxime, IV	1 g vial	5 vials	Organophosphates insecticides
24	Protamine sulfate, IV	10 mg/ml; 5 ml ampoule	1 ampoule	Heparin
25	Pyridoxine, IV	100 mg/ml; 10 ml ampoule	5 ampoules	Isoniazid
26	Sodium bicarbonate, IV	8.4%; 250 ml vial	750 ml (3 vials or 75 ampoules)	Tricyclic antidepressants, serum and urinary alkalization

^a^In cases of treatment for a specific type of poisoning, only the name of the agent is shown.

IV = intravenous; IM = intramuscular; PO = per oral.

The antidote doses and quantities recommended for the initial treatment of a 70 kg adult patient over the first 24 hours after exposure were obtained from the UK guidelines and from standard texts of clinical toxicology.^3^^,^^13^ These were then adapted for the pharmaceutical preparations available on the Brazilian market, as shown in Table 1. This led to 26 antidote options, corresponding to 30 different medicines. For comprehensiveness, from this point onwards, we will call these antidote options simply the “antidotes”.

The availability on the Brazilian market was defined from a previous study and from the authors’ expertise in this field.^8^ In cases in which the antidote was not commercially available, we considered the possibility of extemporaneous preparation or importation.

We elaborated a semi-structured questionnaire in order to gather data on the institutions and antidotes. The person responsible for the pharmacy of the emergency service answered the questionnaire, which was provided on paper.

We did not assess antivenin stockpiles for treating bites/stings caused by native venomous animals. The National Program for Control of Venomous Animals, of the Brazilian Ministry of Health, supplies the stockpiles of antivenin based on the notifications of cases. In Campinas, antivenin stockpiles are available from the regional Poison Control Center.^14^

### Control of bias

To avoid selection bias, we invited all the eligible emergency services to participate, by means of a written invitation and further follow-up calls. We gave assurances of confidentiality by stating that the analysis would be performed in an aggregated manner and that no healthcare service would be negatively exposed through identification in the study.

Two experienced clinical toxicologists (FB, EMDC) reviewed the questionnaire. In this step, the physicians assessed its comprehensiveness and compatibility with Brazilian clinical settings. Although the list of antidotes was based on international guidelines (because of the lack of national standardization), it was adapted to the Brazilian context through the empirical knowledge of these clinicians. Five other healthcare professionals (three physicians and two pharmacists) tested and approved the questionnaire in order to assure understanding (SLSM, ILG, RJV, MY, SMM). The pharmacy director of each service filled out the questionnaire to ensure correctness. These measures were aimed at limiting potential measurement bias.

In cases involving incomplete questionnaire that was returned, we assumed that the antidote in question was not available.

### Statistical methods

The data were entered into an Excel (Microsoft Office 2010) spreadsheet and were analyzed using simple descriptive statistics. We did not perform statistical testing or produce adjusted analyses because of the small sample size.

### Ethical aspects

This study was approved by the institutional Research Ethics Committee of the School of Medical Sciences, State University of Campinas (protocol no. CEP 121/2010). All participants signed a free and informed consent statement.

## RESULTS

Out of the 17 emergency services that were running at the time of the study, 14 (7 public and 7 private) agreed to participate in the study. These emergency services were classified either as pre-hospital (SAMU, n = 1; and UPA, n = 3, 8-21 beds) or as acute hospitals (total of 10, of which: < 50 beds, n = 1; 50-250 beds, n = 8; and > 250 beds, n = 1). The three services not included in this survey were all private: one large and two small hospitals. The reasons for exclusion were refusal (one large hospital); no person responsible for the pharmacy at the time of data collection (one small hospital); and no response and subsequent hospital closure (one small hospital).

Table 2 shows the list of available antidotes according to the emergency service characteristics. All the emergency services stocked 6 out of the 26 recommended antidotes: atropine, sodium bicarbonate, diazepam, phytomenadione, flumazenil and calcium gluconate. Thirteen emergency services also stocked activated charcoal, naloxone and diphenhydramine or biperiden (thus totaling 9/26). None of the emergency departments stocked all of the antidotes surveyed and none had anti-digoxin antibodies, fomepizole, hydroxocobalamin, physostigmine or pralidoxime.

**Table 2.** List of antidotes available in Campinas according to the emergency service profiles

Antidotes^a^	Pre-hospital services		Emergency hospitals			Total n = 14
	SAMU	UPA	Small	Medium	Large	
	(ambulance)	(8-21 beds)	(< 50 beds)	(50-250 beds)	(> 250 beds)	
	n = 1	n = 3	n = 1	n = 8	n = 1	
Atropine	1	3	1	8	1	14
Calcium gluconate 10%	1	3	1	8	1	14
Diazepam	1	3	1	8	1	14
Flumazenil	1	3	1	8	1	14
Phytomenadione (vitamin K)	1	3	1	8	1	14
Sodium bicarbonate	1	3	1	8	1	14
Activated charcoal	0	3	1	8	1	13
Diphenhydramine or biperiden	1	2	1	8	1	13
Naloxone	1	2	1	8	1	13
Acetylcysteine	0	0	1	8	1	10
Dantrolene	0	0	1	8	1	10
Methylene blue	0	0	1	8	1	10
Octreotide	0	0	0	7	1	8
Protamine sulfate	0	0	1	5	1	7
Calcium folinate	0	0	0	3	1	4
Ethanol^b^	0	0	0	3	1	4
Polyethylene glycol 3350	0	0	0	3	1	4
Calcium gluconate gel	0	0	0	0	1	1
Desferrioxamine	0	0	0	0	1	1
Dimercaprol	0	0	0	0	1	1
Pyridoxine	0	0	0	0	1	1
Sodium nitrite and sodium thiosulfate^c^	0	0	0	0	1	1
Glucagon	0	0	0	1	0	1

^a^No emergency service had anti-digoxin antibodies, physostigmine or pralidoxime; ^b^fomepizole was not available; ^c^hydroxocobalamin was not available.

SAMU = Serviço de Atendimento Móvel de Urgência (public emergency ambulance service); UPA = Unidade de Pronto Atendimento (public emergency rooms with a limited capacity for admission, usually less than 24 hours).

Only the hospital at which the regional Poison Control Center is run had antidote stocks close to the recommendation (excluding the five antidotes mentioned earlier and also glucagon); at this service, 100% ethanol was provided instead of fomepizole and sodium nitrite/sodium thiosulfate instead of hydroxocobalamin.

All the emergency hospitals had 12 antidotes with which they were able to start treatment within one hour of admission (diazepam, phytomenadione, flumazenil, calcium gluconate, diphenhydramine or biperiden, sodium bicarbonate, methylene blue acetylcysteine, atropine, activated charcoal, naloxone and dantrolene) (Table 3). To continue the treatment for the first 24 hours in the hospitals, the stockpile was adequate for seven antidotes (diazepam, phytomenadione, flumazenil, calcium gluconate, diphenhydramine or biperiden, sodium bicarbonate and methylene blue).

**Table 3.** Antidotes available at ten emergency departments (emergency hospitals) in the municipality of Campinas, based on the time required for accessing them and the adequacy of the stock for the initial and subsequent 24 hours of treatment for a 70 kg adult patient

Antidotes	Availability in ED (n = 10)	
	< 1 hour	Adequate stock
Diazepam	10	10
Phytomenadione (vitamin K)	10	10
Flumazenil	10	10
Calcium gluconate 10%	10	10
Diphenhydramine or biperiden	10	10
Sodium bicarbonate	10	10
Methylene blue	10	10
Acetylcysteine	10	8
Atropine	10	6
Activated charcoal	10	5
Naloxone	10	5
Dantrolene	10	4
Octreotide	8	7
Protamine sulfate	7	7
Calcium folinate	4	4
Polyethylene glycol 3350	4	2
Ethanol^*^	4	2
Pyridoxine	1	1
Calcium gluconate gel	1	1
Desferrioxamine	1	1
Dimercaprol	1	1
Sodium nitrite and sodium thiosulfate^†^	1	1
Glucagon	1	0

^*^Fomepizole was not available; ^†^hydroxocobalamin was not available. ED = emergency department.

## DISCUSSION

The pre-hospital and hospital emergency services of Campinas have one quarter of the recommended antidotes available for treating poisoning. In the hospital setting, all the emergency departments stocked almost half of the antidotes for starting treatment, but with regard to continuing it for 24 hours, the stockpiles were insufficient for 75% of the antidotes. Only the reference service, which is located in the largest hospital in the city, had an antidote stock that approached the recommended stock profile. This situation is similar to that described in the literature, in which larger hospitals generally have larger, more diversified antidote stocks.^2^^,^^5^^,^^6^

Since no Brazilian recommendations for antidote supply are available, the diagnosis provided by the present study was based on international guidelines. Although the US and UK guidelines for antidote stockpiles can provide a useful starting point, the recommendations may not be realistic from a clinical or economic perspective within our context.

To better address this issue, we compiled a list of the most prevalent poisonings handled by the Campinas Poison Control Center in 2014 (Table 4). Our findings suggest that even the pre-hospital services have sufficient amounts of antidotes to provide the initial medical treatment in most situations. The epidemiological pattern of poison exposure usually has low variation in terms of the agents,^15^ which was the reason why we chose to present this epidemiological data with further updating.

**Table 4.** Most frequent toxic exposures among 5,362 patients followed up by the Campinas Poison Control Center in 2014

Exposures (n = 5,362)*	n	%
Pharmaceuticals		
Sedatives/anticonvulsants	761	14.2
Benzodiazepines	468	8.7
Carbamazepine	113	2.1
Phenobarbital	61	1.1
Antidepressants	403	7.5
Selective serotonin uptake inhibitors	220	4.1
Tricyclic antidepressants	112	2.1
Antipsychotics	236	4.4
Risperidone	45	0.8
Quetiapine	36	0.7
Analgesics and antipyretics	242	4.5
Paracetamol	120	2.2
Dipyrone	96	1.8
Histamine H_1_-receptor antagonists	183	3.4
Venomous animal bites/stings		
Scorpion stings	398	7.4
Snake bites	68	1.3
Spider bites	71	1.3
Caterpillars	69	1.3
Cleaning substances (household)		
Cleansers/detergents/soaps/softeners	366	6.8
Bleaches	139	2.6
Pesticides		
Rodenticides (anticoagulants)	150	2.8
Pyrethroids	122	2.3
Illegal rodenticides *(“chumbinho”)* ^†^	74	1.4
Organophosphates/carbamates	66	1.2
Abused drugs		
Cocaine	125	2.3
Ethanol	91	1.7

*Exposures could be for one or more agents; ^†^ *“Chumbinho”* = illegal rodenticide used in Brazil since the 1990s containing cholinesterase inhibitors, mainly carbamates such as aldicarb.

The following additional limitations of the present survey relating to the source of the data should be noted. The information on local stocks was supplied solely by the pharmacist responsible for the emergency services, with no crosschecking of the data to assess reliability. Some fields in the questionnaire were left blank, which was conservatively considered to represent stock unavailable. Not all of the emergency services in Campinas participated in the study, even though the response rate was above 80%. The study scope was essentially regional, but it represents the first report of antidote availability in emergency services in Brazil.

Making an antidote available does not in itself ensure safety and effectiveness in treating cases of poisoning. Flumazenil, which was found in all stocks, requires judicious evaluation before use for treating benzodiazepine poisoning, since it is contraindicated for concomitant treatment with central nervous system depressors such as tricyclic antidepressants and carbamazepine.^7^^,^^16^ Single-dose activated charcoal can be considered for use in cases involving potentially toxic doses of substances that are adsorbed by activated charcoal; however, there is no evidence that activated charcoal improves the prognosis for patients affected by these substances.^17^ Timely administration is another restriction on the effectiveness of activated charcoal, which should be done within 60 minutes of poison ingestion. Multiple-dose activated charcoal might prevent absorption of some drugs that persist in the gastrointestinal tract (e.g. modified-release preparations), or increase elimination in the postabsorptive phases (enterohepatic or enteroenteric recirculation), and should be considered in cases of ingestion of high doses of carbamazepine, phenobarbital, dapsone, theophylline or quinine, all of which may be life-threatening.^18^

Although dantrolene was stocked by the ten acute hospitals with surgical centers, as recommended by the Brazilian Society of Anesthesiology,^19^ in most cases the stocks held were insufficient.

During the data collection period, none of the emergency departments had any of the first-option antidotes: anti-digoxin antibodies, hydroxocobalamin or fomepizole.^7^^,^^13^ However, some less expensive antidotes may be used as alternatives. For example, sodium nitrite and sodium thiosulfate (methemoglobinizing agents and activators of the rhodanese system), instead of hydroxocobalamin, are also effective for treating cyanide poisoning. However, use of methemoglobinizing agents can be deleterious in poisonings resulting from inhalation of toxic gases containing high concentrations of carbon monoxide and cyanide, especially in urban fires.^7^^,^^13^ In such situations, the first-option emergency antidote is hydroxocobalamin.^7^^,^^13^

Ethanol is an effective alternative to fomepizole for treating methanol/ethylene glycol poisoning. On the other hand, despite the high cost of fomepizole, it is much simpler to use than ethanol.^7^^,^^13^ Fomepizole is particularly useful for treating methanol poisoning caused by massive consumption of adulterated spirits.^20^ Indeed, fomepizole was included in the list of essential antidotes published by the World Health Organization.^21^

Pyridoxine (a first-choice antidote for treating isoniazid poisoning) and dimercaprol (a first-choice antidote for treating acute arsenic poisoning) were stocked in the regional reference service. Since Brazil is among the countries with high incidence and prevalence of tuberculosis,^22^ access to isoniazid is widespread and this increases the risk of toxic exposure. Poisoning due to isoniazid is uncommon, but may result in seizures that are very difficult to control and that improve with high doses of pyridoxine.^13^ Acute poisoning with arsenic is rare nowadays and cases that do occur are generally intentional (attempted suicide and homicide), with serious fulminant complications, hence justifying maintenance of stocks of dimercaprol in the regional reference service.^4^^,^^23^

Although not investigated in this study, other antidotes should be considered for antidote stocks. These antidotes include lipid emulsions for dealing principally with systemic poisoning by local anesthetics;^7^^,^^24^ cyproheptadine for treating serotoninergic syndrome;^25^ continuous infusion of high doses of insulin and glucose as inotropic medicines to counteract poisoning due to myocardial depressors such as β-blockers and calcium channel blockers, instead of glucagon;^7^^,^^26^ and L-carnitine for severe poisoning caused by sodium valproate and valproic acid.^27^ All of these antidotes are stocked in the regional reference service.

Although the cost of some antidotes may appear to be rather high in the context of Brazil’s healthcare system, acquisition of high-cost antidotes could be included in the strategic pharmaceutical assistance component of the Brazilian Ministry of Health, which deals with neglected situations and market availability. Such inclusions would markedly reduce the cost of importation of various antidotes and allow the demands of a greater number of states and municipalities to be met. In this regard, in 2014, at the time when the soccer World Cup was being organized in Brazil, the Campinas Poison Control Center received a supply of hydroxocobalamin sufficient for ten treatments, and the equivalent of four treatments of pralidoxime. Treatment for cyanide poisoning with hydroxocobalamin has been approved by the Brazilian Ministry of Health, with publication of an official guideline and further incorporation in SUS in 2016.^28^^,^^29^ It is valid to say that tragic events, especially the Santa Maria fire in 2013, played an important role in the approval of this antidote in particular. The conclusion of a previous paper in this field remains up-to-date: “Procrastination, fragmentation of responsibilities and improvisation in this area need to be tackled. A policy that anticipates great commotion events or calamity in public health is a pressing need”.^8^

As shown here, this discussion needs to be expanded to define stocks in relation to the immediate needs of emergency rooms and the local epidemiology of poisonings. A recent advance in this regard has been the implementation of an “antidote policy” by the Secretary of Health in the state of Santa Catarina, southern Brazil, based on the strategy adopted in the UK. This policy was formulated in partnership with the local Poison Control Center to incorporate it into the state emergency care network.^30^ This policy and its implementation could provide a basis for creation of similar systems in other Brazilian states or even a basis for a national policy.

In the present survey, we only considered antidotes that are essential for appropriate emergency care of poisoning cases. In low-resource settings, the need for all acute hospitals to stock all of the available recommended antidotes may be unrealistic because of economic constraints. In these situations, strategies such as inter-hospital transfer of antidotes may be effective for treating acute poisonings that require expensive, rarely used antidotes, such as anti-digoxin antibodies. With this system in operation, it would perhaps be irrelevant whether only one or two local hospitals had all of the recommended antidotes. Indeed, these approaches have frequently been used in Campinas, coordinated by the local Poison Control Center, such as the interchange of affordable antidotes like acetylcysteine, ethanol 100%, dimercaprol and antivenins.

## CONCLUSION

In conclusion, the antidote stocks in the emergency services of the municipality of Campinas are incomplete and need to be improved. Our situational awareness can be useful as a starting point in other contexts. For clinical practice, the present findings emphasize the need for an antidote access policy. Further investigations should focus on a national consensus for minimum antidotes and regular stockpile surveys.
